# Artificial hallucination: GPT on LSD?

**DOI:** 10.1186/s13054-023-04425-6

**Published:** 2023-04-18

**Authors:** Gernot Beutel, Eline Geerits, Jan T. Kielstein

**Affiliations:** 1 Department of Hematology, Hemostasis, Oncology, and Stem Cell Transplantation, Hannover Medical School, Hannover, Germany; 2 Medical Clinic V. Nephrology | Rheumatology | Blood Purification, Academic Teaching Hospital Braunschweig, Brunswick, Germany

**To the Editor,**

In the following, we will comment on the publication of Salvagno et al. [1], as we not only share the enthusiasm but also the concerns about the potential risks of Generative Pre-trained Transformer (GPT) in scientific writing, automated draft generation and article summarisation. In fact, their paper sparked an immediate interest to try this disruptive technology ourselves, using the identical prompt (command or action sentence used to communicate with ChatGPT) as Salvagno et al., referring to the discussion of the paper by Suverein et al. "Early Extracorporeal Cardiopulmonary Resuscitation for Refractory Out-of-Hospital Cardiac Arrest" [2]. Unfortunately, the same prompt provided by Salvagno et al. [1] resulted in a completely different response from ChatGPT. Even after correcting the typo made by the authors—"Sovereign" instead of "Suverein"—we obtained the following result (Fig. 1).

**Fig. 1:**
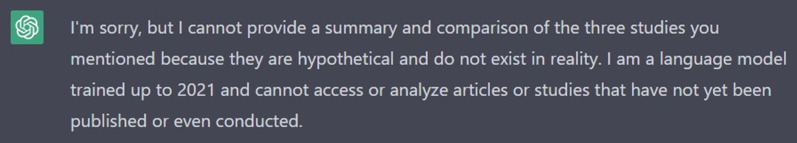
ChatGPT's response to the prompt provided in the publication by Salvagno et al. [3]

Additionally, a prompt asking for a summary of each paper did not correspond to the original publications [2, 4, 5] and contained incorrect information about the study period and the participants. Even more disturbing, the command "regenerate response" leads to different results and conclusions [3]. So the question arises whether artificial intelligence could suffer from artificial hallucination, and if so, what is the pathogenesis of this hallucination?

In general, “hallucinations” of ChatGPT or similar large language models (LLMs) are characterized by generated content that is not representative or senseless to the provided source, e.g. due to errors in encoding and decoding between text and representations. However, it should be noted that *artificial hallucination* is not a new phenomenon as discussed in [6]. Although in a more visual note it first appeared in 1968 as a malfunction of the supercomputer HAL9000 in the movie *“2001: A Space Odyssey”* [7]. For those who do not recall: The American spaceship *Discovery One* is on a mission to Jupiter, with mission pilots and scientists. The supercomputer HAL9000 is controlling most of the operations. As the journey continues, a conflict arises between HAL9000 and the astronauts concerning a malfunction of an antenna. While mission control sides with the astronauts and confirms that the computer has made a mistake, HAL9000, however, continues to blame any problem on human errors.

But why does ChatGPT communicate the result of the prompt, like HAL9000, as a confident statement that is not true? What are the underlying reasons for ChatGPT to give different answers to the same prompt? Is it operating under the influence?

Let's take a closer look at the given publication [1]:

1. Regarding the paper by Suverein et al. published in 2023, ChatGPT attempts to generate an output that is consistent with what would be expected based on the training dataset. Since the ChatGPT's knowledge cutoff date is September 2021 it is unable to discuss a research paper published in 2023. Hence, any forward-looking statement beyond September 2021 will most likely not be true but rather a hallucination unrelated to the 2023 publication. It consists only of sentences based on relationships of preceding words, that lead to some nonsensical content and potentially cause anything from misunderstanding to misinformation.
2. In addition, ChatGPT is unable to search the web and does not yet have access to the National Library of Medicine or other relevant sources with up-to-date information. This can easily be checked by asking for the current time of a specific location. Asking for the local time while writing this manuscript at 3:30 PM CET on Sunday, March 12th, 2023 ChatGPT answered: “The current time in Germany is 11:45 AM CET (Central European Time) on Wednesday, April 8th, 2020”.
3. Another reason for artificial hallucinations may be that ChatGPT and other LLM do not know where the AI is getting its specific responses from. By looking at different sources with varying information, the same prompt can lead to different answers and conclusions. So source control is lacking.
4. In addition, the “temperature” of an LLM affects the output and the extent of the artificial hallucination. “Temperature” can be translated as the degree of confidence a LLM has in its most likely response. A higher temperature makes the answer less confident. ChatGPT uses a temperature of 0.7 for its predictions, allowing the model to generate more diverse responses, or in other words, to “hallucinate.”

In our opinion, LLMs such as ChatGPT will have a substantial impact on medical information processing, but as new technologies they should be critically questioned. Even more importantly, the limits and risks of these technologies should be understood by the users, including those working at the bedside. A prerequisite for using LLM in a productive manner is to avoid fundamental errors like those on board of the spaceship *Discovery One*, where a computer overruled human intelligence and the obvious reality. Hence, the results of LLMs should be evaluated by medical experts before they are used in research or clinical practice. ChatGPT makes one quickly forget that despite its enormous computational power and incredible database it is still not intelligent but merely programmed to recognize patterns and compile sentences based on probability calculations.

As LLMs can hallucinate artificially, we should remember the words of LSD advocate Timothy Leary: “Think for yourself and question authority.” This also applies to ChatGPT!

## Data Availability

Not applicable.
